# Mapping Information Flow in Sensorimotor Networks

**DOI:** 10.1371/journal.pcbi.0020144

**Published:** 2006-10-27

**Authors:** Max Lungarella, Olaf Sporns

**Affiliations:** 1 Department of Mechano-Informatics, The University of Tokyo, Tokyo, Japan; 2 Department of Psychological and Brain Sciences, Indiana University, Bloomington, Indiana, United States of America; University College London, United Kingdom

## Abstract

Biological organisms continuously select and sample information used by their neural structures for perception and action, and for creating coherent cognitive states guiding their autonomous behavior. Information processing, however, is not solely an internal function of the nervous system. Here we show, instead, how sensorimotor interaction and body morphology can induce statistical regularities and information structure in sensory inputs and within the neural control architecture, and how the flow of information between sensors, neural units, and effectors is actively shaped by the interaction with the environment. We analyze sensory and motor data collected from real and simulated robots and reveal the presence of information structure and directed information flow induced by dynamically coupled sensorimotor activity, including effects of motor outputs on sensory inputs. We find that information structure and information flow in sensorimotor networks (a) is spatially and temporally specific; (b) can be affected by learning, and (c) can be affected by changes in body morphology. Our results suggest a fundamental link between physical embeddedness and information, highlighting the effects of embodied interactions on internal (neural) information processing, and illuminating the role of various system components on the generation of behavior.

## Synopsis

How neurons encode and process information is a key problem in computational biology and neuroscience. In this paper, Lungarella and Sporns present a novel application of computational methods to the integration of neural and sensorimotor processes at the systems-level scale. The central result of their study is that sensorimotor interaction and body morphology can induce statistical regularities and information structure in sensory inputs and within the neural control architecture. The informational content of inputs is thus not independent of output, and the authors suggest that neural coding needs to be considered in the context of the “embeddedness” of the organism within its eco-niche. Using robots and nonlinear time-series analysis techniques, they investigate how the flow of information between sensors, neural units, and effectors is actively shaped by interaction with the environment. This study represents a first step towards the development of an explicit quantitative framework that unifies neural and behavioral processes. Such a framework could also shed significant new light on key constraints shaping the evolution and development of nervous systems and their behavioral and cognitive capacities. In addition, it could provide an important design principle to guide the construction of more efficient artificial cognitive systems.

## Introduction

All organisms with nervous systems are physically embedded (embodied) within their respective ecological niche. Their neural structures have evolved to sample and process sensory inputs to create adaptive neural representations, and to select and control motor outputs to position their bodies or to impose changes on the environment. Such sensorimotor activity involves dynamic reciprocal coupling between organism and environment, and a continuous flow of information between sensors, neural units, and effectors. The pattern of information flow defines complex sensorimotor networks, consisting of structured relations and dependencies between sensor, neural, and motor variables. Information structure, such as correlations, redundancies, and invariances in sensory and motor patterns, is critical for adaptivity, robustness, and learning, as well as for enabling action selection, perceptual categorization, and developmental processes [1–3]. Natural stimuli, e.g., visual scenes, contain statistical regularities that are often reflected in the response properties of sensory neurons [4]. The observed match between the structure of sensory inputs and neural responses supports theoretical frameworks suggesting a biological trend towards the development and evolution of optimal neural coding [5,6]. In this paper we examine the hypothesis that statistical regularities in sensory inputs and optimal coding in natural environments are not only the result of the physical properties and statistics of the environment, but can also be induced by the combined action of sensory and motor systems and by body morphology. Building on research in direct and active perception [7–9], and in animate, interactive, and enactive vision [10,11], we adopt the notion that embodied systems actively seek information (stimuli) while engaging in behavior. We employ physical and simulated robots that serve as models of embodied organisms, sharing their embeddedness and dynamical coupling, while being significantly easier to manipulate and monitor [12]. In previous work, we found that coordinated and dynamically coupled sensorimotor activity induces quantifiable changes in sensory information, including decreased entropy, increased mutual information, integration, and complexity within specific regions of sensory space [13,14]. In this paper, we demonstrate the existence of networks of directed information flow between specific sensory, neural, and motor variables. These networks are dependent on the degree of sensorimotor coupling between the embodied organism and its environment, on experience-dependent plasticity and learning, and on morphological features of the body. Our adoption of a quantitative framework based on information theory allows, in principle, for an investigation of these effects across a broad range of living systems, and may provide a novel link between neural coding, behavioral dynamics, and the evolution of morphology.

**Figure 1:**
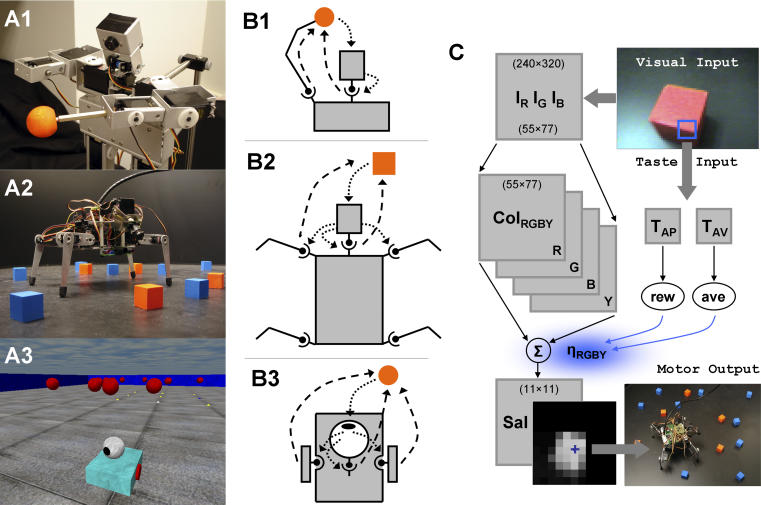
Robots, Sensorimotor Interactions, and Neural Control Architecture (A1) *Roboto* has a total of 14 DOF, five of which are used in the current set of experiments. Note the head-mounted CCD camera, the pan-tilt head system (2 DOF), and the moveable left arm with shoulder, elbow, and wrist joints (3 DOF). The object is a red ball (1.25 inches diameter) attached to the tip of the last joint. (A2) *Strider* has a total of 14 DOF, with four legs of 3 DOF each and 2 DOF in the pan-tilt head system. Objects are red and blue blocks (1 inch cubes). *Strider* is situated in an environmental enclosure with black walls. (A3) *Madame* has 4 DOF, with 2 DOF in the pan-tilt system and 2 DOF for the wheels, which are both located on an axis vertical to the main body axis. The environment is a square arena bounded by blue walls containing 20 red-colored floating spheres. (B1) *Roboto* engages in sensorimotor interactions via the head system and arm movements; sensory → motor (dotted arrows), motor → sensory (dashed arrows). (B2) *Strider* engages in sensorimotor interactions via the head system, as well as via steering signals generated by the head and transmitted to the four legs. (B3) *Madame*'s behavior consists of a series of approaches to colored objects and ovations. Fixations to the objects are maintained by independent action of head and body. (C) Neural control architecture. The architecture common to all robots is composed of color image arrays *I_R_, I_G_, I_B_,* color- intensity map***Col*** *_RGBY_*, and saliency map *Sal* (see text for details). The peak of the saliency map (blue cross) determines the pan-tilt camera motion and body steering. In addition, *Strider*'s neural system contains a value system with taste sensory inputs relayed via a virtual taste sensor (blue square in visual image) to taste neurons (*T_AP,AV_*), which in turn generates reward and aversiveness signals (rew, ave). These signals are used to modulate the strengths of the saliency factors *η_RGBY_* (see text for details).

In what follows, we identify a set of fundamental mechanisms (involving sensorimotor interaction and body morphology) that support and complement biological information processing carried out by nervous systems. We introduce a set of measures designed to detect information structure and directed information flow between coupled systems, such as brain, body, and environment. In particular, we opt for a “model-free” approach to data analysis, and use mutual information and transfer entropy [15] to discriminate nondirected and directed components of sensorimotor coupling. We find that information structure and information flow can be mapped between a variety of sensory and motor variables recorded from three morphologically different robotic platforms (a humanoid robot, a mobile quadruped, and a mobile wheeled robot), each of which reveals a specific aspect of information flow in embodied systems (Figure 1). First, using the humanoid robot controlled by a saliency-based visual system, we show that the degree of sensorimotor coupling is reflected in the information structure and the strength of information flow between sensory, neural, and motor variables. Second, we illustrate how experience-dependent learning and plasticity can affect the directed transfer of information in sensorimotor networks. Specifically, changes in the neural system of the mobile quadruped robot, which depend on reward and aversiveness for particular types of objects, give rise to changing patterns of information flow between sensory, neural, and motor variables. Third, we demonstrate the effect of changes in body morphology on information flow, by varying the arrangement of photoreceptors in the simulated retina of the one-eyed mobile wheeled robot. In the final section, we make predictions and develop further hypotheses.

## Results

We analyze several sensory and motor variables collected from three different robotic platforms and reveal the presence of causal structure induced by dynamically coupled sensorimotor activity. Causal linkages between sensory and motor states are spatially and temporally specific, and are sensitive to changing environments and movement strategies.

### Effects of Sensorimotor Coupling on Information Structure and Flow

We evaluated the contribution of sensorimotor coupling to all informational measures by comparing two experimental conditions, one in which sensorimotor coupling was undisturbed and one in which sensorimotor coupling was disrupted—we refer to these two conditions as “fov” and “rnd,” respectively. In condition fov, all sensory, neural, and motor dynamics unfolded without intervention in real time. In condition rnd, a previously recorded motor signal was substituted, resulting in motor activity that was not driven by actual real-time sensory inputs. This enforced dissociation between sensory and motor data was designed to disrupt sensorimotor coupling, while leaving intact the statistical patterns within both sensory and motor domains. Differences in informational measures between these two conditions can be attributed to the presence or absence of coupling between sensory and motor streams. Thus, condition rnd represents an interventional or perturbational approach designed to discern patterns of information flow caused by sensorimotor coupling.

**Figure 2:**
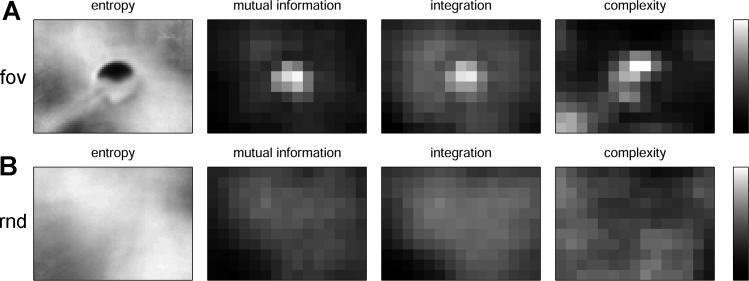
Information Structure in Sensorimotor Data Obtained from *Roboto* Data represents average profiles obtained from five runs per condition (1,000 time steps each). Resulting maps show (from left to right) entropy, mutual information, integration, and complexity for “fov” (A) and “rnd” (B) conditions. Gray scale ranges (at right) are [2 4] bits (entropy), [0.35 0.95] bits (mutual information), [23 48] bits (integration), and [0.4 0.65] bits (complexity).

Figure 2 shows maps of entropy, mutual information, integration, and complexity for data collected from array *I_R_* in *Roboto*. If the sensorimotor interaction was undisturbed (condition fov, Figure 2A), we observed increased cumulative intensity of the color red near the center of the visual field (unpublished data), as well as decreased entropy and increased mutual information, integration, and complexity. All measures exhibited significantly weaker effects if sensorimotor interaction was disrupted (condition rnd, Figure 2B). Very similar informational patterns were observed for neural activation states of *Sal,* as well as for *I_R_, I_G_, I_B_,* and *Sal* in *Strider* (unpublished data). These results were entirely consistent with those reported earlier for a different robotic pan-tilt platform tracking salient stimuli in color movies [13]. While both platforms shared similar active vision control architectures, their body morphologies were significantly different, as was the nature of their visual stimulation.

**Figure 3:**
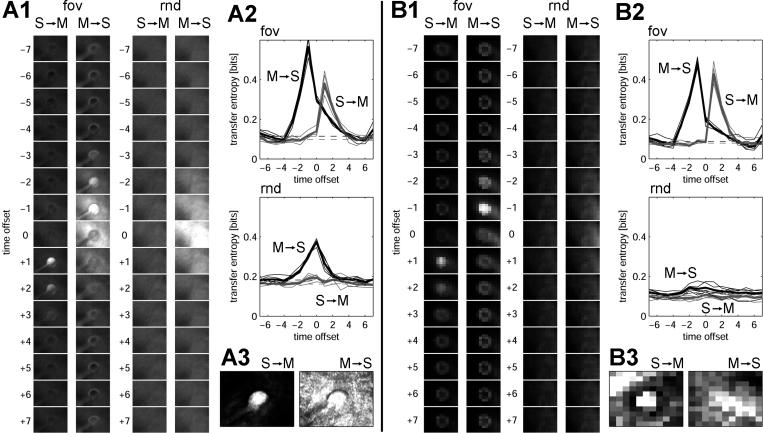
Information Flow (Transfer Entropy) between Sensory Input, Neural Representation of Saliency, and Motor Variables in *Roboto* (A1) Transfer entropy between array *I_R_* (variable S) and pan-tilt amplitude (variable M). Series of plots show maps of transfer entropy from S to M (S → M) and from M to S (M → S) over visual space (55 × 77 pixels), calculated for offsets between −7 (“M leading S”) and +7 (“S leading M”) time steps. Plots show data for conditions “fov” and “rnd.” The gray scale ranges from 0.0 to 0.5 bits (for all plots in panels A1 and B1). (A2) Curves show transfer entropy for five individual runs (thin lines) as well as the average over five runs (thick lines) between the single central pixel of array *I_R_* (S) and pan-tilt amplitude (M), for directions M → S (black) and S → M (gray). (A3) z-Score maps of significant image regions (plotted between z = 0 and z = 6). The z-scores are expressed as number of standard deviations above background at time offset +1 (S → M) and −1 (M → S). Mean and standard deviation of background is calculated from transfer entropy values at maximal time delays (−7,+7 time steps). (B) All three panels have the same format as (A), but the neural activations of the saliency map *Sal* are substituted as variable S (11 × 11 neural units).

Figure 3 summarizes results obtained from an analysis of directed information flow using transfer entropy, performed on the same datasets used for the noncausal analyses shown in Figure 2. The introduction of variable time offsets between sensory (S) and motor (M) time-series data allowed us to plot causal relations between these variables across all time delays (Figure 3A1 and 3B1). When examining the relation between visual inputs (S, array *I_R_,* Figure 3A1) and the amplitude of pan-tilt head movements (M) for condition fov, we found positive transfer entropy in the direction S → M at offsets of +1 and (with decreasing magnitude) for offsets bigger than +1. No transfer entropy was found for offsets less than or equal to zero. In the reverse direction, we found transfer entropy in the direction M → S when M preceded S by at least one time step (time offset = −1) with a falloff towards more negative offsets. For condition rnd, transfer entropy was diminished if not eliminated, in accordance with the experimentally introduced disruption of causal interactions between sensory and motor time series. Residual transfer entropy in the direction M → S persisted in condition rnd, as pan-tilt head movements continued to cause displacements of the visual scene, albeit decoupled from those of the red object. The analysis also revealed the presence (for all time offsets) of elevated transfer entropy near the edges of the stimulus object (“ring-like” structures in Figure 3A and 3B), indicating that discontinuities in the visual image (e.g., at object boundaries) produce state transitions that are more effective in driving changes in motor variables. Figure 3A2 shows plots of transfer entropy across all offsets for the center of array *I_R_*. By comparing the transfer entropy at or near zero time offset to baseline values (z-score maps for transfer entropy; Figure 3A3), we also provided a statistical estimate of image regions that either exerted significant causal effects on motor states (S → M) or were causally affected by motor states (M → S). We found that only the surface representation of the red object caused head displacement, which in turn caused displacement of the entire visual scene (including background). Figure 3B utilized the activity pattern of the saliency map, a neural variable, as the sensory (S) time series. Similar patterns of causality were revealed, with peak transfer entropies that were equal to, if not greater in magnitude than, those obtained analyzing data from *I_R_*.

Peak transfer entropies depended on a variety of factors, including some key parameters in the behavioral pattern. We varied the “jump frequency” i.e., the frequency with which the object in *Roboto*'s hand was translated to a new randomly chosen position. This “jump” could not be predicted by the head system and acted like a large environmentally driven perturbation that elicited corrective action by the pan-tilt unit to maintain foveation. Peak transfer entropies declined as these jumps became less frequent; peak *T*(*S → M*) was 0.29, 0.28, 0.23, 0.12, 0.05 bits, for jump frequencies of 5, 10, 20, 50, and 100 time steps (n = 5 runs). No significant transfer entropy was measured if the object was not moved at all and always remained foveated. Transfer entropy was zero if no changes occurred. This result indicates that transfer entropy increases with the amount of environmental changes causing behavioral responses—even if the perception–action loop is unperturbed (as for condition fov).

**Figure 4:**
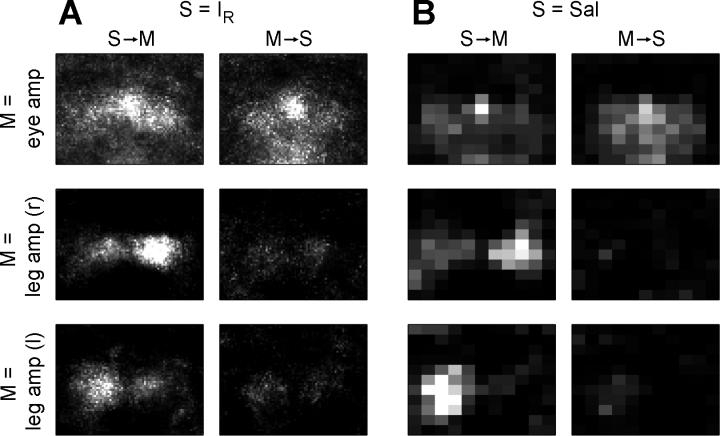
Information Flow (Transfer Entropy) in *Strider* Data is shown as z-score maps, plotted between z = 0 and z = 6, for a variety of sensory and motor variables. The z-scores are expressed as number of standard deviations above background, calculated as for Figure 3. (A) Transfer entropy between S = red intensity map *I_R_* (55 × 77), and M = eye (pan-tilt) amplitude (top), M = right-leg amplitude (middle), and M = left-leg amplitude (bottom). (B) Transfer entropy for S = downsampled saliency map *Sal* (11 × 11). M variables correspond to those in (A).

Estimates of information structure (entropy, mutual information, integration, and complexity) as well as transfer entropy maps for sensory and motor variables were obtained from a second morphologically and behaviorally different robotic platform, the quadruped *Strider*. Distributions of information across the visual field were comparable to those obtained with *Roboto* (unpublished data), indicating increased levels of information structure near the visual fovea and for behaviorally salient feature maps. Summaries of spatial profiles (z-score maps) for transfer entropy obtained from *Strider* are displayed in Figure 4. While considerably more noisy than maps obtained from *Roboto,* profiles for *T*(*S → M*) and *T*(*M → S*) reveal similar causal relations between *I_R_, Sal,* and pan-tilt amplitude, with peaks for transfer entropy near the center of the visual field. Plots of *T*(*S → M*) and *T*(*M → S*) between sensory maps and leg movement amplitudes display peaks that are laterally displaced, reflecting the efficacy of steering input relayed from laterally located visual targets via the head system to the legs. For example, visual targets on the right side resulted in decreased movement amplitudes of the legs on the right side of the body axis, a causal connection that is retrieved via estimation of transfer entropy between the appropriate sensorimotor variables.

### Effects of Learning on Information Flow

**Figure 5:**
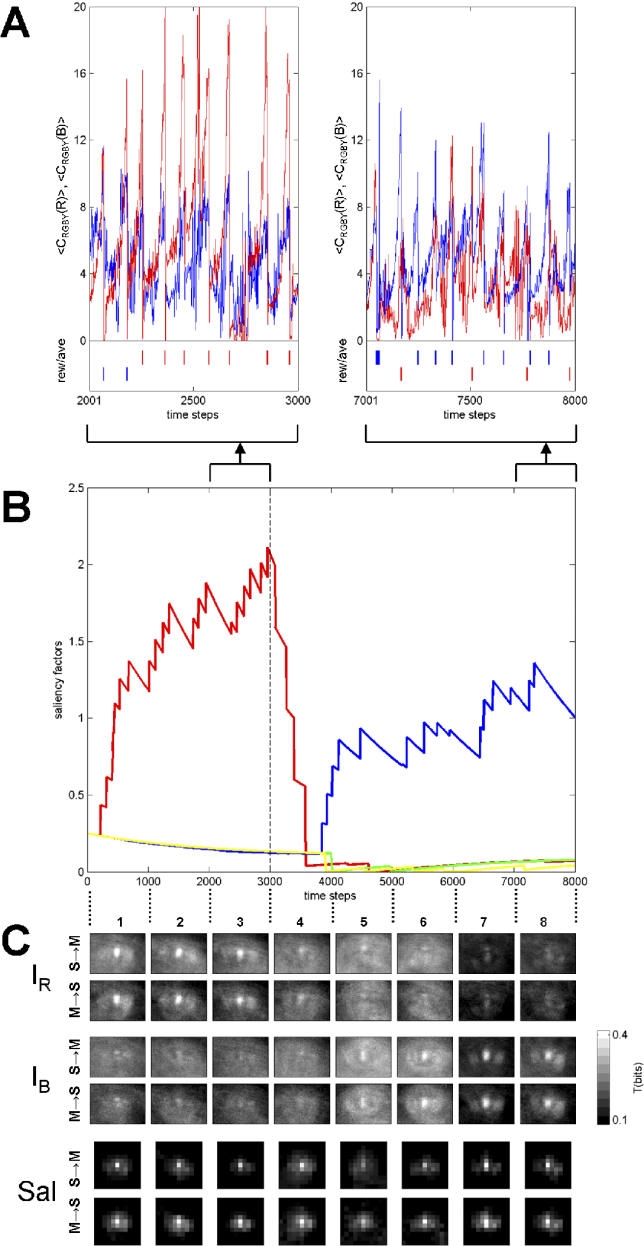
Changes in Behaviour, Saliency Factors, and Information Flow (Transfer Entropy) in *Strider* Data are from a single representative experiment, collected over 8,000 time steps. *Strider* navigated though its enclosure and approaching and “tasting” salient objects. Saliency (contingency) was under experimental control, and was switched at t = 3,000 from red = rewarding and blue = aversive to red = aversive and blue = rewarding. (A) Traces of average activation levels in color-selective maps ***Col*** *_RGBY_*, for red and blue (green and yellow are not shown for clarity), sampled between t = 2,001 and 3,000 (left plot) and t = 7,001 and 8,000 (right plot). Raster plot at the bottom gives corresponding rewarding (rew, top trace) and aversive (ave, bottom trace) events, with the color of the plot corresponding to the dominant color present in the center of the visual field at the time of the reward/aversive signal. (B) Traces of saliency factors *η_RGBY_* over time. Note reversal of red/blue at the time of contingency switch (t = 3,000). (C) Transfer entropy maps for sensory variables S = *I_R_* (top rows), S = *I_B_* (middle rows), and S = *Sal* (bottom rows). M = eye (pan-tilt) amplitude throughout. Gray scale ranges from 0.1 to 0.4 bits.

To illustrate the effects of learning on patterns of information flow, we included a value system as part of the neural architecture of *Strider* (Figure 1C). Based on changes in reward and aversiveness, the value system was capable of modulating saliency factors that, in turn, were used to compute the activation profile of the saliency map controlling head movements. Close encounters with objects resulted in the deployment of a virtual taste sensor. Positive changes in this sensor's activation triggered reward (appetitive taste) and aversive (aversive taste) signals (Figure 1C). Reward and aversive signals, in turn, modulated saliency factors such that encounters of rewarding objects tended to increase the saliency factor for that object's color, while encounters of aversive objects had the opposite effect. If the saliency distribution of objects in the environment changed over time (e.g., previously rewarding objects became aversive, and vice versa), the system adapted through changes in the saliency factors. As saliency factors changed, different objects became capable of “capturing attention” and causing approach or foveation behavior. These changes in saliency and attention could be monitored by recording behavioral and neural data. Figure 5A shows sample traces of average activities of the color maps and the value system collected in the course of a representative experiment lasting 8,000 time steps (approximately 13 minutes of real time). Activations of color maps increased as objects were approached and peaked around the time of object encounter. Sensing of taste triggered a value signal (either rewarding or aversive) coupled with visual inputs relaying the object's color. The comparison of two time segments, before and after a switch in color-taste contingency was made, documents a switch in behavior from approach of red objects to approach of blue objects, as well as changed reward/aversiveness profiles. Figure 5B displays the time course of saliency factors during the same experiment as shown in Figure 5A. The reversal of reward/aversiveness at time step t = 3,000 is accompanied by a reversal of saliency factors for red and blue, due to the modulatory actions of the value system.

Changes also occurred in sensorimotor information flow, i.e., transfer entropy. Transfer entropy maps obtained from a representative experiment (matching Figure 5A and 5B) are shown in Figure 5C. Initially (from t = 1 to t = 3,000), red objects were rewarding, leading to an increase in the corresponding saliency factor *η_R_,* accompanied by a strong flow of information from red visual sensors near the fovea to the pan-tilt head system. Peak transfer entropy from sensory to motor variables was *T*(*S → M*) = 0.449 bits, while the peak value in the reverse direction was *T*(*M → S*) = 0.447 bits. As reward was switched from red to blue objects (at t = 3,000), the saliency factors adjusted, with *η_B_* becoming dominant around t = 4,000. This adaptive change was reflected in a decrease of information flow emanating from red sensors and an increase of information flow from blue sensors to pan-tilt motors with a peak value of *T*(*S → M*) = 0.412 bits. Peak values for transfer entropy from the neural saliency map to motors remained high throughout the experiment, as the map continually drove foveation behavior, even while its afferent connections adapted to changes in the environment.

### Effects of Morphology on Information Flow

Body shape, limb articulation, as well as the position and density of sensors on the body surface have a large impact on sensory and motor capabilities of an organism. For instance, theoretical and experimental studies demonstrate that the distribution of retinal cells (e.g., cones, rods, ganglion cells) impacts the coding and transmission of the retinal image to higher levels in the visual pathway [16–18]. To take a specific case, can the morphology of visual sensors affect visuo–motor information flow? We recall that the retina of most biological eyes is a variable resolution (space–variant) sensor: the mosaic formed by the photoreceptors (cones and rods) across the retinal surface is inhomogeneous, yielding a spatial resolution that varies across the visual field [19,20]. In primates, the density of cones (used for high acuity vision) is typically greatest in the center (fovea) and falls off with retinal eccentricity (angular distance from the center of gaze). This morphological arrangement simultaneously enables high acuity of some parts of the visual field (achieved by appropriate head/eye movements) and a wide field of view, without requiring an enormous number of sensing elements and processing resources.

**Figure 6:**
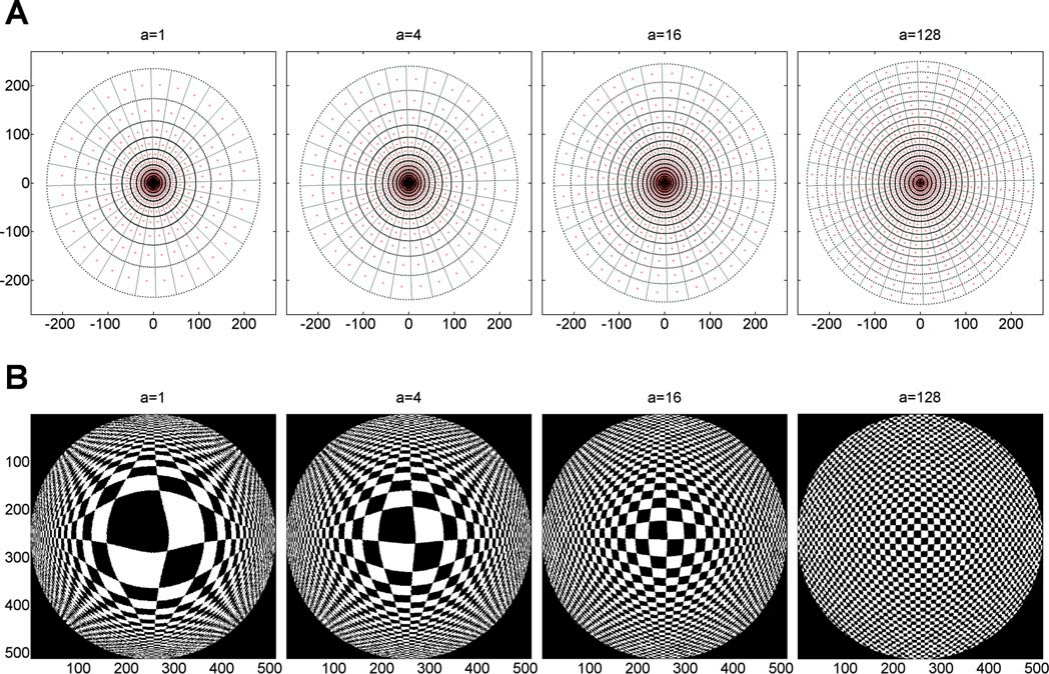
Illustration of the Retino-Cortical Mapping Process (A) Iso-density contours of photoreceptors (red crosses) for four selected values of the parameter *a*. The photoreceptors are arranged as 18 concentric rings. The mapping “template” is composed of nonoverlapping 32-ring sectors (receptive fields) formed by the intersection of rays originating at the center of the retina. The value of the photoreceptor is the average of the intensities of the photoreceptors (pixels) within the receptive field's boundary. (B) Inverse mapping of “cortical” images back to the retinal (input) domain. The cortical magnification effect is due to inhomogeneous distribution of photoreceptors. A “Cartesian” visual image (here, a checkerboard; 512 × 512 pixels) is mapped onto a “cortical” plane. Mapping is dependent on the linear parameter *a*. The foveal part of visual field is magnified, that is, a larger piece of cortical area is devoted to processing.

Body morphology is shaped in the course of evolution and development and is hard to manipulate systematically in either animals or physical robots. Our approach was to bypass this experimental difficulty by using a simulated mobile robot (*Madame*). Of all possible implementations of visual sensors, in *Madame* we implemented variants of retinal morphologies with a “log-polar” distribution of photoreceptors (here, only cones) [21–23]. The log-polar geometry has been shown to model accurately the topographical (retino–cortical) mapping of retinal cells (cones or ganglion cells) to the geniculate body and the striate cortex (area V1) [16,24]. We define the mapping from the “Cartesian retina” (x,y) onto the “cortical” plane (u,v) as the following coordinate change: *u*(*r*,*θ*) = *k*log(*r*/*a* + 1), *v*(*r*, *θ*) = *θ* where *k* is a normalization constant, the parameter *a* determines the density distribution of the retinal cells, and polar coordinates (*r*,*θ*) are used to replace Cartesian ones (*x*,*y*) in the retina: *r* = 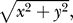 *θ* = tan^−1^(*y*/*x*). A possible implementation of this arrangement is shown in Figure 6A, where a constant number of photoreceptors (represented by crosses) is arranged so as to give rise to an increase of the cells' spacings with respect to the distance from the central point of the structure. The mapping “template” is composed of nonoverlapping ring sectors (receptive fields) formed by the intersection of rays originating at the center of the retina. The photoreceptors' activities are calculated as the average of the intensities of the photoreceptors within their receptive fields. The larger number of receptors in the foveal part of the retina leads to a visual magnification in the cortical plane (Figure 6B). Magnified areas correspond to receptors that are proportionally more important than others requiring more accurate information processing [25]. The visual magnification factor is defined as the derivative of the mapping *k*/(*r* + *a*), and has a magnitude inversely proportional to distance from the center of the retina. Note that the derivative is radially symmetric, and roughly inversely proportional to the retinal eccentricity, approximating the retinotopic structure of the cat, owl monkey, rhesus monkey, and human visual cortex [16].

**Figure 7:**
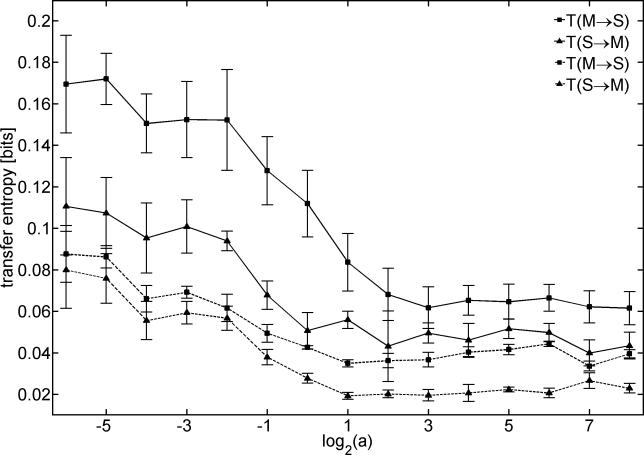
Effect of Retinal Morphology on Information Flow Transfer entropy between sensor (S) and motor variables (M) as a function of parameter *a* in *Madame*. The linear parameter *a* determines the distribution of the photoreceptors in the retina and thus the eye morphology. Transfer entropy is calculated for every pixel of a visual region (S; 6 × 6 pixel patch) and the difference between left and right wheel speed (M; angular velocity). Squares show information flow from M to S; triangles indicate information flow from S to M. Pixels were selected from a central visual region (continuous lines) and a peripheral region (dashed lines). Data in all graphs are averages of five representative experiments consisting of 4,096 samples each, error bars show standard deviations.

Here, we not only show that morphology shapes information flow but we also provide a quantitative measure for the amount of flow (the behavior of the robot was qualitatively the same throughout all experiments). Figure 7 displays transfer entropy for values of the parameter *a* = 2*^k^*(*k* ∈ [−5,8]) evaluated as the average over both a region of the visual field and multiple experimental runs. The transfer entropy was calculated by averaging the transfer entropy between every pixel of a central (and noncentral) 6 × 6 pixel patch (variable S) and the difference between angular speed of left and right wheel (variable M). The error bars in Figure 7 denote standard deviations calculated for five runs of 4,096 samples each. Invariably, *T*(*M → S*) was larger than *T*(*S → M*) (for both central and noncentral visual patches), i.e., motor variables (e.g., difference between left and right angular speed) were more effective in driving sensor variables (e.g., red color intensity map) than vice versa. A striking result is that the information flow *T*(*M → S*) and *T*(*S → M*) for *a* < 0.25 is larger than for *a* > 2, with a transition between the two regions characterized by a clear inflection in the profile. This inflection is most likely a function of the size of the object on the robot's retina. Such “apparent” (or relative) size is used to regulate the distance of the robot from the object: the closer the artificial creature is to the object, the larger the object looms in front of it, and the more the creature slows down. The “causal” effect is more evident for lower values of *a* because the visual magnification factor is larger. Clear numerical differences can be also seen in the standard deviation. As a result of the visual magnification effect and in accord with our intuitions, the standard deviation for larger values of *a* is lower. Notably, the standard deviation for *T*(*M → S*) for *a* = {0.125,0.25,0.5,1.0} is significantly larger than for *T*(*S → M*). By contrast, for *a* > 2 and *a* < 0.125, the standard deviations for the two conditions are of comparable magnitude. By increasing the visual area over which the transfer entropy was calculated (up to patches of 12 × 12 central pixels), we observed no significant change in the resulting plots (unpublished data). In the case of noncentral visual patches, the graph flattens and the inflection is less pronounced; also the standard deviations are smaller. Given that the visual magnification is inversely proportional to the distance from the center, peripheral areas of the retina are less effective in driving the motor variables, and, vice versa, the effect of movements is less pronounced in the periphery.

These results indicate that eye morphology can affect information structure (here, mutual information) and information flow (here, transfer entropy), and how such effects of morphology can be quantified. Our findings are consistent with the hypothesis that morphology has an effect on information measures capturing statistical interactions and dynamic dependencies between variables.

## Discussion

As organisms interact with their environment, their sensory inputs are transformed into motor outputs and their motor outputs determine what is sensed next. The continuous and dynamic coupling between sensory, neural, and motor variables defines sensorimotor networks that describe the informational embedding of organisms within their ecological niches at multiple time scales. The comparison of the relative influence such variables exert on each other helps extract (functional and structural) patterns of interaction between the networks' elements that may support biological information processing. In this paper we provide a quantitative framework for how to map these sensorimotor networks, which by using mutual information and transfer entropy allows capture of undirected and directed exchanges of information (information flow) between sensory, neural, and motor variables in three physically embedded (embodied) systems. Our central hypothesis is that sensorimotor interaction and morphological structure induce information structure in the sensory input and neural system, promoting information processing and flow between sensory input and motor output. We find that information flow in sensorimotor networks is (a) *quantifiable and variable in magnitude;* (b) *temporally specific,* i.e., restricted to short temporal delays between sensory and motor time series; (c) *spatially specific,* i.e., restricted to specific portions of the visual input capable of driving motor responses; (d) *modifiable with experience,* e.g., in the course of value-dependent learning of stimulus–response contingencies; and (e) *dependent upon morphology,* e.g., the density and distribution of visual sensors. Our results are robust with respect to the details of the sensory and motor systems employed, and hold across several different robotic platforms (stationary and mobile, simulated and real) and a range of sensory and motor variables.

Information structure created by sensorimotor interactions is evident from a variety of informational measures including basic functionals such as entropy and mutual information, which have been discussed in detail elsewhere [13]. In this paper, we placed special emphasis on directed information transfer (information flow) between sensory, neural, and motor variables, and not on static correlations or undirected (shared) information. A variety of measures of directed information transfer (and “causal dependency”) are available; they rely on the use of univariate and multivariate time-series analysis and embedding techniques [26–29], probabilistic graphical models (e.g., Bayesian networks [30]), and perturbation analysis [31]. Based on the results of a comparison study [26], we chose transfer entropy [15] as our measure of information flow because it makes minimal assumptions about the dynamics of the time series, captures linear and nonlinear effects, and is numerically stable even for reasonably small sample sizes (1,000 samples). We stress that to infer “causal dependency” from mere time-series data is problematic, due to the often nonlinear, transient, and noisy quality of the data and due to uncertainty introduced by the potential existence of unobserved variables or hidden common sources. In general, approaches based on observational quantities alone are not able to disclose a full causal picture of the system, and interventional (or perturbational) techniques (e.g., [31,32]) will ultimately be needed to provide a truly causal description of sensorimotor and neural networks. This inherent weakness of time-series−based measures does not undermine their “heuristic” usefulness in detecting directed information transfer and mapping sensorimotor networks if care is taken in the design of the experimental setup and the selection of the observables and the state space. For example, our comparison between unperturbed and perturbed experimental conditions (fov and rnd) has allowed the identification of directed relationships in sensorimotor networks caused by sensorimotor coupling. In other approaches, measures based on time-series analysis have been applied to real and simulated neural datasets [33–35], revealing patterns of information flow (and causal dependencies) within extended neural systems in the course of behavioral or cognitive tasks. The link between causal networks and behavior has been addressed in [36], demonstrating that rich adaptive behavior displays a higher density of causal interactions in neural networks, as well as a stronger flow of information from sensory input to motor output.

Various approaches linking information structure and neural processing have been suggested on the basis of information theory considerations. These include modelling frameworks for effective information transmission [37,38], efficient and sparse coding [5,6,39], visual attention [40], extraction of behaviorally relevant stimulus properties [41], and information processing in sensorimotor systems [42,43]. Each of the proposed approaches predicts specific transformations of stimulus representations along the processing hierarchy. Our work complements these studies in several ways: (1) The extension of the information theory approach to sensorimotor networks in embodied systems naturally captures the effects of motor outputs on sensory inputs, an aspect often neglected in work focusing only on information processing in neural systems. In this paper we identified ways in which sensorimotor coupling can generate additional information that may promote more efficient neural coding. (2) Techniques that map directed information flow can simultaneously be applied to sensory, motor, and neural variables. Although we focused mostly on sensory and motor variables while mapping sensorimotor networks, such networks readily extend across all hierarchical levels of neural processing. (3) The morphology of an embodied system can have significant effects on its information processing capacity. We tested the hypothesis that sensor morphology (here, the arrangement of photoreceptors in a simulated retina) influences the flow of information in a sensorimotor system.

The last point in the previous paragraph supports the notion of a quantitative link between the morphology of the retina and a computational principle of “optimal flow of information.” Given a fixed number of photosensitive elements, their space-variant arrangement maximizes the information gathered, even more so in a system engaged in a sensorimotor interaction, e.g., foveation behavior. If the photoreceptors were uniformly distributed in the retina, those in the periphery would be underutilized; also, fewer photoreceptors would be in the fovea, yielding (on average) lower spatial resolution, and resulting in less accurate estimates of object locations. Such non-uniformity at the receptor level is mirrored by non-uniformity at the cortical level in a topology-preserving fashion, that is, nearby parts of the sensory world are processed in nearby locations in the cortex. There has been some work on deriving such topology-preserving maps through the principles of uniform cortical information density [25] and entropy maximization [44]. We argue here that in a sensorimotor system, the rate of information transfer is maximized at the receptor stage if the probability distribution of target objects on the retina is adapted to the local photoreceptor density (a morphological property), and that this can be achieved through appropriate system–environment interaction, e.g., foveation, saccades, or adequate hand movements [45]. A further implication of our findings relates to the possible role of early visual processing for the learning of causal relationships between stimuli. It has been shown, for instance, that the receptive fields of retinal ganglion cells produce efficient (predictive) coding of the average visual scene [17,46]. We propose that such coding also depends on the local arrangement of the receptors and on the spatial frequencies encountered during the organism's lifetime.

In conclusion, our results highlight the fundamental importance of embodied interactions and body morphology in biological information processing, supporting a conceptual view of cognition that is based on the interplay between physical and information processes. In line with this view, most theories of embodied cognition are built around the notion that intelligent behavior and cognitive processes are the result of the continuous interaction and the reciprocal causal influence of brain, body, and environment [47–51]. According to these theories, it is the complex and dynamic interaction of neural processing, bodily action, and environmental forces that forms the basis of real-time adaptive response. Our work represents a step towards the development of an explicit quantitative framework that restores the unity of body and brain on the basis of their informational dependencies. Such a framework could also shed significant new light on key constraints shaping the evolution and development of nervous systems and their behavioral and cognitive capacities. In addition, a quantitative framework for information flow in embodied systems could provide an important design principle [14,52] to guide the construction of more efficient artificial cognitive systems.

## Materials and Methods

### Robots.

We used three morphologically and behaviorally different robotic platforms, a fixed miniature humanoid named *Roboto* (Figure 1A1), a mobile quadruped named *Strider* (Figure 1A2), and a simulated mobile robot with wheels named *Madame* (Figure 1A3).

*Roboto.* For the present experiments we used five of *Roboto*'s 14 kinematic degrees of freedom (DOF), three in the left arm (shoulder, elbow, and wrist), and two in the head system (pan and tilt), which was equipped with a centrally mounted CCD camera. A red object (visual target) was connected to the tip of the arm's most distal link and the arm was moved in a preprogrammed pattern. Initially the arm and object were positioned directly in front of *Roboto,* in view of the CCD camera. Every ten time steps, the arm was abruptly moved to a randomly chosen new position (a “jump”), selected within a range of the workspace of the individual joints, resulting in a displacement of the object relative to the head. Motor actions of the head were under visual control (see below), and displacement of the visual target resulted in foveation, with the robot tracking the position of the object as the arm was moved (Figure 1B1).

*Strider.* While *Roboto* was mounted on a pedestal, *Strider* (Figure 1A2) was fully mobile and situated within a tub-like environmental enclosure (~1 m diameter) containing a number of stationary colored cubes. Two front-mounted infrared sensors were used for wall avoidance. Locomotion was generated by rhythmic movement of the four legs (12 DOF), using ipsilateral and contralateral phase coupling between the legs [53]. The head system contained two DOF (pan, tilt) and was similar in construction and identical in terms of neural control to that of *Roboto* (see below). Motor actions of the head system were under visual control, resulting in foveation of colored objects. The position of the head system was relayed to the locomotion controller to steer *Strider* by modulating the movement amplitudes of two of the legs. This amplitude modulation resulted in gradual orientation of the body axis towards the object, while fixation was maintained by the independent action of the head system (Figure 1B2). The resulting behavior was a series of approaches to colored objects, each lasting for about 20 time steps, with intermittent periods of searching for new targets while navigating through the environment. All experiments were carried out with 12 red and 12 blue objects, initially positioned at random throughout the environment.

*Madame.* The third robot was implemented in simulation. It consisted of a mobile two-wheeled platform (of length L) equipped with seven proximity sensors for obstacle avoidance and a pan-tilt camera unit. The pan and the tilt angles were constrained to vary in an angular interval of 60° relative to the robot's midline. The environment was a square arena bounded by blue walls (of length 40L) containing 20 red-colored floating spheres (of diameter 0.3L) placed in random locations. The elevation of the spheres was L and was affected by a small amount of Gaussian noise. The spheres were relocated to a new position (not too far from the previous position) every 300 time steps. Neural control of the head system was identical to that used in *Roboto* and *Strider* (see below). Similar to *Strider*, *Madame*'s behavior consisted of a series of approaches to colored objects and foveations with intermittent periods of search while moving through the environment. Fixation to the objects was maintained by independent action of head and body (Figure 1B3 illustrates the sensorimotor links).

### Neural control architecture.

All three robots used an active vision system (Figure 1C) designed to direct attention—and thus processing resources—to particular locations in space according to their behavioral relevance or saliency, here encoded exclusively by color feature maps [13]. The design of the color and saliency system closely followed the model of Itti et al. [54]. The sampled raw visual images were first luminance-scaled according to standard formulae, and then used to compute color-opponent maps for red, green, blue, and yellow (*R, G, B,* and *Y*). Subsequently, an opponent threshold was applied, followed by a “winner-take-all” mechanism resulting in four color-intensity maps *Col_RGBY_(R), Col_RGBY_(G), Col_RGBY_(B), and Col_RGBY_(Y),* which recorded the pixel-wise thresholded intensity of the dominant colors *R*, *G*, *B,* and *Y*. A color-saliency map normalized to [0, 1] was created by linear summation of the individual intensity maps as *Sal* = ***η**_RGBY_ ·* ***Col*** *_RGBY_*, where ***η**_RGBY_* = [*η_R_*,*η_G_*,*η_B_*,*η_Y_*] and ***Col*** *_RGBY_* = [*Col_RGBY_*(*R*),*Col_RGBY_*(*G*),*Col_RGBY_*(*B*),*Col_RGBY_*(*Y*)], and by scaling to the global maximum. The saliency factors *η_R_, η_G_, η_B_,* and *η_Y_* encoded the relative saliency of each of the four color components. In the experiments with *Roboto* and *Madame*, *η_R_* was set to one, and all other saliency factors were set to zero, which resulted in a strong preference of the active vision system for the color red. In the experiments with *Strider,* saliency factors were modified dependent upon experience (see below). Once derived, the color saliency map was “block-averaged” to yield a map *Sal* with lower spatial resolution, whose global maximum determined the spatial location to which eye/camera movements were directed. The spatial coordinates of the maximum were then transformed into servo motor commands relayed to the pan-tilt motors moving the camera and to the servo motors driving the legs (*Strider*) and wheels (*Madame*). Camera motion resulted in lateral image shifts and a repositioning of the saliency profile. For stationary objects, camera motion stabilized quickly to direct gaze toward the maximum of the saliency map (foveation).

In the experiments with *Strider,* the saliency factors were subject to experience-dependent plasticity. Plastic changes were under the control of a value system [55,56] capable of influencing the coupling between ***Col*** *_RGBY_* and *Sal* in the presence of changes in innately salient sensory stimulation, analogous to biological neuromodulatory systems (e.g., [57]). Innately salient sensory inputs were modelled as “virtual taste,” sampled through a small virtual tastepad attached below the camera. Whenever the camera pointed downward, indicative of close approach and foveation of a target object, the virtual tastepad was activated. Taste inputs were either appetitive (*T_AP_*) or aversive (*T_AV_*), depending on the color of the object. Color-taste associations were under experimental control and varied between red-appetitive/blue-aversive and red-aversive/blue-appetitive. The level of appetitive and aversive taste input was transformed into a rewarding [rew(t)] or aversive neural signal [ave(t)], respectively, according to:

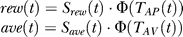


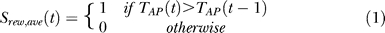
with Φ(·) denoting a standard sigmoidal function used to scale *T_AP_* and *T_AV_* to the interval [0, 1]. The saliency factors *η_RGBY_* were adjusted by means of the following equation:

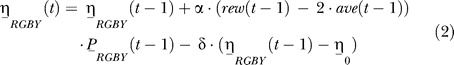



***P*** *_RGBY_* corresponded to a binary representation of the activation of the color intensity map ***Col*** *_RGBY_* in the center of the visual field (e.g., a red object generated ***P*** *_RGBY_* = [1 0 0 0]). The incremental learning rate *α* was set to 0.2, and the decay rate *δ* = 0.0005, with ***η*** *_0_* = [0.1, 0.1, 0.1, 0.1] and |***η*** *_RGBY_* (t)| ≥ 0 at all times. At the beginning of every run, the saliency factors were initialized as ***η*** *_RGBY_*(0) = [0.25,0.25,0.25,0.25]. During experience, positive changes in the taste input (i.e., the onset of rewarding or aversive sensation) generated phasic and graded reward/aversive signals that were used to increase (in the case of reward) or decrease (in the case of aversiveness) the saliency factors.

### Informational measures.

We note that the meaning of the term information differs depending on the particular context. Here, information is used in the Shannon sense, that is, to quantify statistical patterns in observed variables. We applied a set of five informational measures, all of them fundamentally based on Shannon entropy [58,59]. Four of these measures (entropy, mutual information, integration, and complexity) capture statistical regularities between random variables without taking into account temporal precedence. These measures as well as some of the details of their computational derivation are discussed in more detail in [13]. To estimate directed information flow, we used a measure developed for time-series analysis, transfer entropy [15].

*Shannon entropy.* Given a time series *x_t_* that can assume N states, entropy provides a measure of the average uncertainty, or information, calculated from the state probability distribution according to:

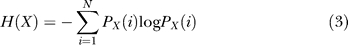
where *P_X_*(*i*) is the probability of *x_t_* being in the i^th^ state. Entropy is maximal if all states occur with equal probability (maximal disorder or uncertainty), while deviations from equal probability result in lowered entropy (increased order and decreased uncertainty).

*Mutual information.* Mutual information is a general measure of association between two or more random variables, naturally encompassing both linear and nonlinear dependencies. The formal definition of mutual information in terms of single and joint state probability distributions is

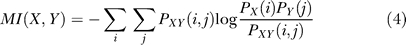
If *X* and *Y* are two statistically independent random variables, *P_XY_*(*i*, *j*) = *P_x_*(*i*)*P_Y_*(*j*) and *MI*(*X*,*Y*) = 0. In a sense, mutual information quantifies the error we make in assuming *X* and *Y* as independent variables, i.e., any statistical dependence between *X* and *Y* yields *MI*(*X*,*Y*) > 0. In general, statistical dependency as measured by mutual information is insufficient to disclose directed interactions (e.g., causal relationships) between *X* and *Y,* or between *Y* and *X,* thus requiring the use of special techniques (see below).

*Integration.* Integration (or multi-information; [60]) is the multivariate generalization of mutual information and captures the total amount of statistical dependency among a set of random variables *X_i_* forming elements of a system ***X* =** {*X_i_* }. Integration [61] is defined as the difference between the individual entropies of the elements and their joint entropy:

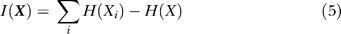
As for mutual information, if all elements *X_i_* are statistically independent, *I(* ***X*** *)* = 0. Any amount of statistical dependence leads to *I(* ***X*** *)* > 0.

*Complexity.* If a system ***X*** has positive integration, i.e., some amount of statistical dependence, we may ask how such statistical dependence is distributed within the system. If the system consists of locally segregated and globally integrated components, we would expect to find statistical dependence among units at specific spatial scales. A system combining local and global structure has high complexity:

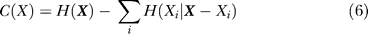
where *H*(*X_i_*|***X*** – *X_i_*) is the conditional entropy of one element *X_i_* given the complement ***X*** – *X_i_* composing the rest of the system. Previous studies (e.g., [62]) have shown that complexity is high for systems that effectively combine local and global order, e.g., systems that are both functionally segregated and functionally integrated. On the other hand, complexity is low for systems that are entirely random or entirely uniform.

*Transfer entropy.* In addition to these noncausal informational measures, we used a measure that aims at extracting directed flow or transfer of information (also referred to as “causal dependency”) between time series, called transfer entropy [15]. Given two time series *x_t_* and *y_t_,* transfer entropy essentially quantifies the deviation from the generalized Markov property: *p*(*x_t_*_+1_|*x_t_*) = *p*(*x_t_*_+1_|*x_t_*,*y_t_*), where *p* denotes the transition probability. If the deviation from a generalized Markov process is small, then the state of ***Y*** can be assumed to have no (or little) relevance on the transition probabilities of system ***X***. If the deviation is large, however, then the assumption of a Markov process is not valid. The incorrectness of the assumption can be expressed by the transfer entropy, formulated as a specific version of the Kullback-Leibler entropy [15]:

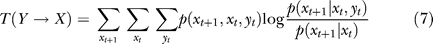
where the sums are over all amplitude states, and the index *T*(*Y → X*) indicates the influence of ***Y*** on ***X***. The transfer entropy is explicitly nonsymmetric under the exchange of ***X*** and ***Y***—a similar expression exists for *T*(*X → Y*)—and can thus be used to detect the directed exchange of information (e.g., information flow, or causal influence) between two systems. As a special case of the conditional Kullback-Leibler entropy, transfer entropy is non-negative, any information flow between the two systems resulting in *T* > 0. In the absence of information flow, i.e., if the state of system ***Y*** has no influence on the transition probabilities of system ***X*** *,* or if ***X*** and ***Y*** are completely synchronized, *T*(*Y → X*) = 0 bit.

In summary, applied to sensory, neural, and motor datasets, entropy quantifies the average uncertainty (or self-information) about the state of individual elements, while mutual information measures the statistical dependency between two elements. Integration (or multi-information) serves as the multivariate extension of mutual information, capturing the degree to which two or more elements share information. The degree to which individual elements are specialized (representing statistical independence) while also sharing information (through global interdependence) is captured by complexity. Transfer entropy is designed to detect “directed” information exchange or coupling between two elements or parts of a system.

### Data collection.

In *Roboto* and *Strider,* visual sensory data was collected at a frame rate of approximately 10 Hz by head-mounted CCD video cameras and separated into red, green, and blue components. The raw visual images were sampled at a resolution of 240 × 320 pixels, and downsampled to 55 × 77 pixels (arrays *I_R_, I_G_, I_B_*). Motor data was collected as motor commands were issued to the robot's servos and saved as angular positions for each separate servo motor. Servo positions were issued and recorded at a resolution of 256 steps per approximately 100° rotation. Time series of individual motor positions were transformed into movement amplitudes and directions by temporal differencing. For *Roboto* and *Strider,* we collected data from five runs per experimental condition. For *Roboto,* all runs had a length of 1,000 time steps (approximately 100 s). For *Strider*, runs involving learning had a length of 8,000 time steps (approximately 800 s). For *Madame*, the raw visual images were sampled at a resolution of 80 × 80 pixels and downsampled to 40 × 40 pixels. As for both physical robots, sensor and motor data (speed of left and right wheel and angular displacement of pan and tilt motor) were collected as motor commands were issued to the agent's actuators. We collected data from five runs with a length of 4,096 simulation steps each (note that although the sampling frequency cannot be meaningfully expressed as updates/s (in Hz), it can be taken to be equivalent to the sampling frequencies of the two physical robots, 10 Hz).

On a more general note, the simulation time step (sampling period) needs to “match” the behavioral/neural time scale. Here, we chose the minimal possible sampling period, which is one time step. There is no possible sampling period below one time step (as time steps cannot be subdivided). Having designed the systems, we know that this time scale matters, because it is the time scale at which sensors sample the environment and at which motors change position.

### Data analysis.

All numerical computations for data analysis were carried out in Matlab (Mathworks, http://www.mathworks.com). To calculate entropy and mutual information, datasamples were discretized (16 states, 4 bits) to allow robust estimates of probability distributions. Mutual information, integration, and complexity were calculated from differenced datasets, i.e., the original time series *x_t_* was replaced with its first-order temporal derivative, *y_t_* = *x_t_* – *x_t_*_−1_. Differenced datasets remove trends while exhibiting improved stationarity. In addition, the use of the first temporal derivative mimics the sensitivity of visual neurons to spatial and temporal changes in visual inputs, resulting in more stable representations of object properties especially in the presence of object motion. To estimate integration and complexity, we used statistical formulae that allow the calculation of entropies from the covariance matrix, under the assumption that these covariances were generated by a stationary Gaussian random process with zero mean and unit variance [58]. All differenced datasamples were examined for Gaussian state distributions (by fitting state histograms) as well as stationarity (by ensuring stable means and standard deviations across time). Nonstationary datasets were excluded from analysis. To calculate transfer entropy, time series were discretized to eight states (3 bits) and joint probabilities and conditional probabilities were approximated by means of kernel density estimation. As in [13], we chose a step kernel. Temporal delays across time series were introduced by shifting one time series relative to the other, thus allowing the evaluation of directed relationships across variable time offsets (or delays). Such delays could potentially be introduced due to the discrete nature of the updating of the control architecture and due to the temporal persistence of sensory and motor states. All results reported in this paper were qualitatively robust with respect to the specific choice of state space over a broad range of discretization (32 to four states).

## Abbreviations

DOF: degrees of freedom
